# Disease-Associated Mutations That Alter the RNA Structural Ensemble

**DOI:** 10.1371/journal.pgen.1001074

**Published:** 2010-08-19

**Authors:** Matthew Halvorsen, Joshua S. Martin, Sam Broadaway, Alain Laederach

**Affiliations:** 1 Biomedical Sciences Department, University at Albany, Albany, New York, United States of America; 2 Developmental Genetics and Bioinformatics, Wadsworth Center, Albany, New York, United States of America; National Institute of Genetics, Japan

## Abstract

Genome-wide association studies (GWAS) often identify disease-associated mutations in intergenic and non-coding regions of the genome. Given the high percentage of the human genome that is transcribed, we postulate that for some observed associations the disease phenotype is caused by a structural rearrangement in a regulatory region of the RNA transcript. To identify such mutations, we have performed a genome-wide analysis of all known disease-associated Single Nucleotide Polymorphisms (SNPs) from the Human Gene Mutation Database (HGMD) that map to the untranslated regions (UTRs) of a gene. Rather than using minimum free energy approaches (e.g. mFold), we use a partition function calculation that takes into consideration the ensemble of possible RNA conformations for a given sequence. We identified in the human genome disease-associated SNPs that significantly alter the global conformation of the UTR to which they map. For six disease-states (Hyperferritinemia Cataract Syndrome, β-Thalassemia, Cartilage-Hair Hypoplasia, Retinoblastoma, Chronic Obstructive Pulmonary Disease (COPD), and Hypertension), we identified multiple SNPs in UTRs that alter the mRNA structural ensemble of the associated genes. Using a Boltzmann sampling procedure for sub-optimal RNA structures, we are able to characterize and visualize the nature of the conformational changes induced by the disease-associated mutations in the structural ensemble. We observe in several cases (specifically the 5′ UTRs of FTL and RB1) SNP–induced conformational changes analogous to those observed in bacterial regulatory Riboswitches when specific ligands bind. We propose that the UTR and SNP combinations we identify constitute a “RiboSNitch,” that is a regulatory RNA in which a specific SNP has a structural consequence that results in a disease phenotype. Our SNPfold algorithm can help identify RiboSNitches by leveraging GWAS data and an analysis of the mRNA structural ensemble.

## Author Summary

Genome-wide association studies identify mutations in the human genome that correlate with a particular disease. It is common to find mutations associated with disease in the non-coding region of the genome. These non-coding mutations are more difficult to interpret at a molecular level, because they do not affect the protein sequence. In this study, we analyze disease-associated mutations in non-coding regions of our genome in the context of their structural effect on the message of genetic information in our cells, Ribonucleic Acid (RNA). We focus in particular on the regulatory parts of our genes known as untranslated regions. We find that certain disease-associated mutations in these regulatory untranslated regions have a significant effect on the structure of the RNA message. We call these elements “RiboSNitches,” because they act like switches turning on and off genes, but are caused by Single Nucleotide Polymorphisms (SNPs), which are single point mutations in our genome. The RiboSNitches we identify are potentially a new class of pharmaceutical targets, as it is possible to change the structure of RNA with small drug-like molecules.

## Introduction

Genome-Wide Association Studies (GWAS) pinpoint mutations associated to a disease state with single nucleotide precision [1]–[4]. In some cases, the molecular cause of the disease is evident from the mutation data alone. For example, if the mutation results in a premature stop codon, the production of a truncated protein is the cause for the disease [5]. In a majority of cases, however, it is difficult to identify the molecular cause of the disease from the GWAS data alone [3], [6]–[11]. This is especially true when associations are identified in non-coding and intergenic regions of the genome [10], [11]. Since a majority of the human genome is non-coding and intergenic, it is not surprising that many GWAS studies are finding disease associations in such regions [12]–[14]. In this study we aim to evaluate the role of mutation induced structural changes in regulatory RNAs of the human genome and their consequence on the disease state.

The central role of RNA as a major regulator of genetic networks in the cell is now well established [15]. Furthermore, it is estimated that up to 95% of the human genome is transcribed, suggesting that a majority of mutations are transferred to the transcriptome [1]. This study focuses on the potential structural consequences of disease-associated mutations on the RNA transcriptome, in particular single nucleotide polymorphisms (SNPs) in the 5′ and 3′ UTRs of genes. UTRs are the regulatory elements of genes, acting as controllers of translation and RNA decay, as well as targets for RNA interference (RNAi) [16]–[18]. Since UTRs are readily transcribed, play a central role in post-transcriptional regulation, and are integral to the mature mRNA, they present an ideal starting point for studying the potential structure/function relationships of disease-associated mutations on the transcriptome.

Unlike highly structured RNAs such as self splicing introns [19], Riboswitches [20], and the Ribosome [21], the UTRs of mRNAs are not generally evolved to adopt single, well-defined structures. Instead they adopt an ensemble of conformations best described by a partition function, which is defined as the probabilities of all possible base-pairs [22]–[24]. Most mutations in an RNA only have local effects on the structural ensemble. A small subset of mutations, however, have a large and global effect [22]. If a disease-associated mutation belongs to the latter, it can suggest a role for RNA structure in the molecular mechanism of the disease. We make several assumptions in this study, which will be borne out by the data presented below. These assumptions are:

1. Certain human disease states are caused by mutation induced conformational changes in transcribed, regulatory RNA molecules. If a disease-associated mutation causes a large change in the ensemble of RNA structure, this suggests RNA conformational change as a potential molecular cause of the disease.
2. Large regulatory RNAs generally adopt multiple conformations and it is critical to consider how mutations affect this ensemble rather than just the minimum free energy structure [25].
3. A majority (>95%) of mutations result in only small, local changes in the structure of an RNA.
4. The same phenotype (disease) can be caused by different mutations with varying degrees of effect on overall RNA ensemble structure. A global analysis of the structural consequences of all disease-associated mutations on a regulatory RNA can pinpoint the regulatory region of the RNA.

In this study we investigate known disease associated SNPs that map to non-coding UTR regions of the human genome with respect to their effect on the ensemble RNA structure. We identify disease states in which the associated SNPs significantly alter the RNA structural ensemble of the UTR. This analysis provides insight into the potential molecular causes of several genetic disorders including Hyperferritinemia-cataract syndrome [26], β-Thalassemia [27], [28], and Chronic Obstructive Pulmonary Disease (COPD) [29], [30]. More importantly, our analysis reveals the extent to which SNPs affect RNA structure, and the nature of those effects in disease-states.

## Results

### Ensemble RNA structural analysis

**Figure 1:**
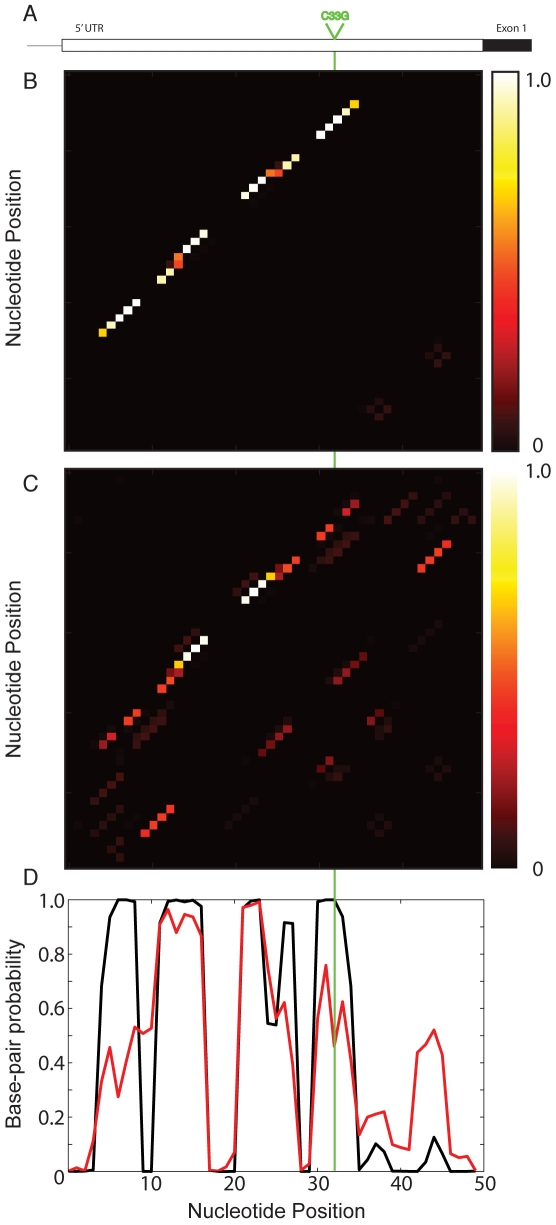
Partition function analysis of the C33G SNP in the 5′ UTR of HBB associated with β-Thalassemia [**28**]. (A) Schematic representation of the HBB gene, showing the 5′ UTR and the start of the first exon (black). The C33G SNP position is indicated in green. (B) Partition function heat map for the wild-type (non-diseased) 5′UTR RNA illustrating base-pair probabilities. The rectangle to the right of the heat map is a legend, with zero probability being black and a probability of one colored white. (C) Partition function heat map for the HBB 5′ UTR RNA with the diseased G allele at position 33. The appearance of alternative structures is apparent when compared to the non-diseased C allele above. (D) Nucleotide base-pair probability (or accessibility) of the HBB 5′ UTR for the wild-type (non-diseased, black) and mutant (disease-associated) RNA (red). The base-pair probability is computed by summing the rows (or columns) of the partition function. We compute the Pearson correlation coefficient between the wild-type (black) and disease-associated mutation (red) lines to quantify the change in the structural ensemble caused by mutation. In this case, we compute a Pearson correlation coefficient of 0.797 for the C33G mutation.

We first consider the C33G SNP in the 5′ UTR of the HBB (β-globin) gene, which is associated with β-Thalassemia [31], [32] to illustrate the basic premise of our methodology. The SNP is not located near any transcription, translation start or stop sites (Figure 1A). A recent study demonstrated that the C33G mutation (replacing C33 with a G) has a negligible effect on mRNA transcriptional levels [33]. A possible cause for the disease state is therefore a conformational change in the RNA structure. In Figure 1B, we show the result of a partition function calculation for the wild-type (non-diseased) “C” allele of the UTR. Unlike traditional Minimum Free Energy calculations (MFE) that predict a single low energy structure of the RNA, the partition function computes the probability of pairing for all possible base-pairs including potential pseudoknots [22]–[24]. The partition function therefore is a representation of the RNA structural ensemble, i.e. all possible RNA structures [22]. Since whole UTRs are generally not evolved to adopt a single well defined structure, the partition function illustrated in Figure 1B is a more accurate representation of the RNA's structural ensemble than the single structure obtained by traditional MFE computations such as mFold [23].

We choose to highlight the HBB 5′ UTR and the C33G SNP associated with β-Thalassemia [31], [32] because of the difference in the partition functions illustrated in Figure 1B and 1C. The partition function calculation using the mutant sequence (replacing C33 with a G) is dramatically altered by this single SNP, suggesting a significant change in the overall structural ensemble of the UTR RNA. In Figure 1D, we compute the base accessibility (i.e. the probability of the base being paired) by summing the base-pair probabilities down the columns of the partition function. When we compare the base-pairing probabilities for the wild type (C33 non-diseased allele, black line) with the disease-associated mutation (G33, red line), we see that specific bases show large changes in nucleotide accessibility while others remain unaffected by this mutation.

### Evaluating the significance of a change in the RNA structural ensemble

For the purposes of this study, we are particularly interested in identifying disease-associated SNPs like C33G in the HBB 5′ UTR that have a significant effect on the RNA structural ensemble as defined by the partition function calculation. We quantify the overall structural effect of a mutation on an RNA by computing the Pearson correlation coefficient between the wild-type and diseased base-pair probabilities (black and red lines, Figure 1D). For the C33G mutant we determine a WT/mutation correlation coefficient of 0.797 (Table 1). This simple calculation allows us to quantitatively describe the overall rearrangement in the structural ensemble of the RNA caused by the disease-associated mutation.

**Table 1:** Disease states and phenotypes in which two or more associated SNPs were found to alter the structural ensemble of the RNA.

Disease/phenotype	Gene	HGMD Accession	UTR	NTs	SNP	Corr. Coeff	p-val	ref.	Motifs^1^	RBP^2^ Binding	dbSNP^3^ ref. ID
Alteration of plasma zymogen TAFI concentration	CPB2	CR080756	3	427	T310A	0.640	0.001	[45]	uORF, MBE, PAS	-	rs1087
				453	T336A	0.826	0.094				
Chronic obstructive pulmonary disease	SERPINA1	CR061339	5	533	C116T	0.664	0.013	[34]	uORFs	-	rs8004738*
				554		0.784	0.033		uORFs	-	
				551		0.777	0.040		uORFs	-	
Retinoblastoma	RB1	CR961736	5	166	G17C	0.679	0.014	[65]	IRES	ELAVL1	-
		CR086248			G18T	0.766	0.098	[66]			-
Hyperferritinemia Cataract Syndrome	FTL	CR011064	5	199	C14G	0.673	0.020	[67]	IRE	-	-
		CR061336			A56T	0.713	0.042	[68]			-
		CR061334			T22G	0.766	0.065	[68]			-
		CR031001			C10T	0.792	0.072	[69]			-
Cartilage-Hair Hypoplasia	RMRP	CR063415	nc-RNA	265	T252G	0.738	0.029	[70]	-	-	-
		CR054274			G40A	0.761	0.047				-
		CR054268			G182T	0.801	0.083				-
β-Thalassemia	HBB	CR900265	3	132	A11G	0.794	0.033	[27]	PAS	-	rs63751128
		CR984119			C47G	0.799	0.038	[71]			-
		CR014260			T110G	0.815	0.045	[72]			-
		CR880076			A113G	0.841	0.071	[73]			rs33985472
		CR961734	5	50	C33G	0.797	0.040	[28]	-	-	rs34135787
	HBD	CR075247	5	195	G66A	0.771	0.070	[74]	uORF, MBE	-	-
Hypertension	AGT	CR971935	5	508	G465A	0.694	0.051	[75]	uORFs	ELAVL1PABPC1	rs5051*
		CR973338			A451C	0.765	0.089	[76]			rs5050*

1 Structural and sequence motifs identified in mRNA UTRs using UTRScan [62].

2 RNA Binding Protein as determined by RIP-chip [46].

3 dbSNP reference IDs for common variants. A star (*) indicates LD data is available and reported in Figure S5.

**Figure 2:**
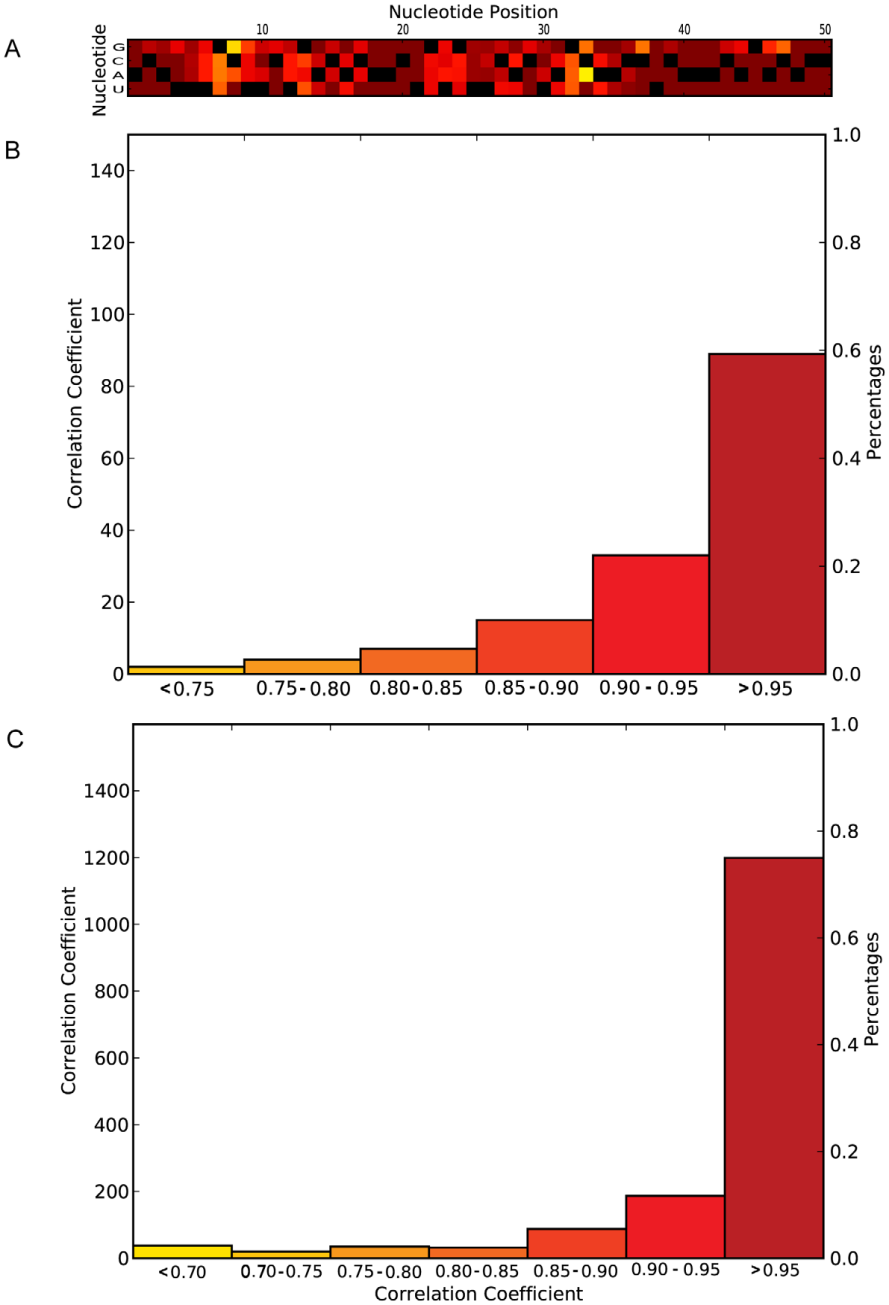
Comprehensive single mutation analysis of the HBB 5′ UTR to determine the significance of the observed rearrangement in the structural ensemble caused by mutation. (A) Heat map diagram illustrating the Pearson correlation coefficients for all possible mutations in the HBB 5′ UTR. The heatmap color scheme is identical to that used in Figure 1B and 1C. The four rows on the diagram each indicate a different nucleotide (A, C, G, or U) while each column represents a position in the UTR. The wild-type sequence is indicated with black boxes. Only a few mutations (e.g. C33A, C10A) including the C33G result in small (<0.8) Pearson correlation coefficients. (B) Histogram of Pearson correlation coefficient values for all 150 possible mutations in the HBB 5′ UTR. A majority of mutations (<95%) have correlation coefficients greater than 0.9. We use these calculations to estimate a p-value for the significance of the observed structural change in the ensemble. (C) Similar histogram for all mutations in the 5′ UTR of the SERPINA1 gene where C116U is associated with Chronic Obstructive Pulmonary Disease (COPD) [34]. The distribution of Pearson correlation coefficient values gets steeper with longer RNAs (the 5′ UTR of SERPINA1 is 533 nucleotides long).

The Pearson correlation coefficient as computed above provides a quantitative measure of the overall change in the partition function caused by a mutation. However, based on this single calculation, it is difficult to determine the significance of the structural change. We compute Pearson correlation coefficients for all 150 possible single nucleotide mutations (the HBB 5′ UTR is 50 nucleotides in length) and illustrate their values as a heat map in Figure 2A. This result illustrates that a majority of mutations in the HBB 5′ UTR only have small effects (Pearson correlation coefficient >0.95) on the structural ensemble. To better illustrate this point, we plot in Figure 2B a histogram of Pearson correlation coefficients for all single nucleotide mutations of HBB.

The distribution of Pearson correlation coefficients is dependent on both the sequence and its length. This is illustrated in Figure 2C where we plot the distribution of Pearson correlation coefficients for the 1599 mutations in the 5′ UTR of SERPINA1 (serpin peptidase inhibitor, clade A (α-1 antiproteinase, antitrypsin), member 1, which is 533 nucleotides in length), where the C116U SNP is associated with COPD [34]. The two distributions are clearly different and these results suggest a straightforward approach for comparing the extent of conformational change caused by a SNP in an RNA. The C33G mutation in the HBB 5′ UTR has the sixth lowest correlation coefficient out of the 150 possible mutations and we therefore compute a p-value of 6/150 = 0.04 for this SNP (Table 1). Similarly, the C116U mutation in the 5′ UTR of SERPINA1 results in a Pearson correlation coefficient of 0.664 and this yields a p-value of 21/1599 = 0.013. This simple calculation allows us to compare the effects on SNPs on different UTRs and thus rank order the disease-associated SNPs in the Human genome with respect to the significance of the structural rearrangement they induce.

### Genomic scan of all known disease-associated SNPs in HGMD

We analyzed a total of 514 disease-associated SNPS in 350 UTRs and non-coding RNAs from the HGMD (Human Gene Mutation Database) [35], [36]. HGMD is a curated database that records the results of published GWAS and other disease association studies [35]. This database is unique in that it provides flanking sequence for a majority of its entries, allowing us to automatically validate the location of SNPs within UTRs using the latest human genome annotations [37], [38]. Of the 350 RNAs we analyzed, 206 were 5′ UTRs, 132 were 3′ UTRs and 12 were non-coding RNAs. The SNPs we analyzed map only to the untranslated regions of mature mRNA and are at least 10 nt away from any transcription or translation start or stop sites. Furthermore, the HGMD annotation stores SNPs associated with alternative splicing in a separate table, which we did not include in our analysis. Our data therefore represents a comprehensive subset of known disease-associated mutations within mRNA UTRs that are not expected to directly affect splicing, translation or transcription through sequence variation. We chose to perform our analysis on this particular subset of disease-associated SNPs to maximize our chances of finding disease states where RNA structural rearrangements are likely to be causative in the association. We map in Figure S5 all SNPs in strong LD (Linkage Disequilibrium, R^2^>0.9) for common variants identified in Table 1.

Our results are presented in Table 1 and in Table S1. We report on all the disease-associated SNPs that alter RNA structure with a p-value<0.1. We therefore report the top 10 percent of disease-associated SNPs in regulatory non-coding RNA that alter their RNA structural ensemble within the human genome. The disease-states reported in Table 1 are particularly interesting to this study, as they potentially offer mechanistic insight into how RNA structural rearrangement can affect gene regulation and lead to disease. We begin our analysis of SNP induced RNA conformational change by considering the four SNPs associated with Hyperferritinemia Cataract Syndrome listed in Table 1.

### Hyperferritinemia Cataract Syndrome

We identify four SNPs in the 5′ UTR of the FTL (ferritin light chain) gene that significantly affect the RNA structural ensemble (Table 1) and that are associated with Hyperferritinemia Cataract Syndrome. The FTL gene encodes the Ferritin light chain protein, and deregulation of this gene leads to the disease phenotype [39]. Recent studies on the regulation of FTL have revealed an Iron Response Element (IRE) in the 5′ UTR to which a regulatory Iron Response Protein (IRP) binds [26], [39]. The IRE is an RNA hairpin and mutations in the 5′ UTR disrupt the structure of the IRE and thus alter the binding affinity of the IRP, leading to aberrant FTL regulation [26]. This type of regulatory system is precisely what we aim to identify with our genomic analysis.

**Figure 3:**
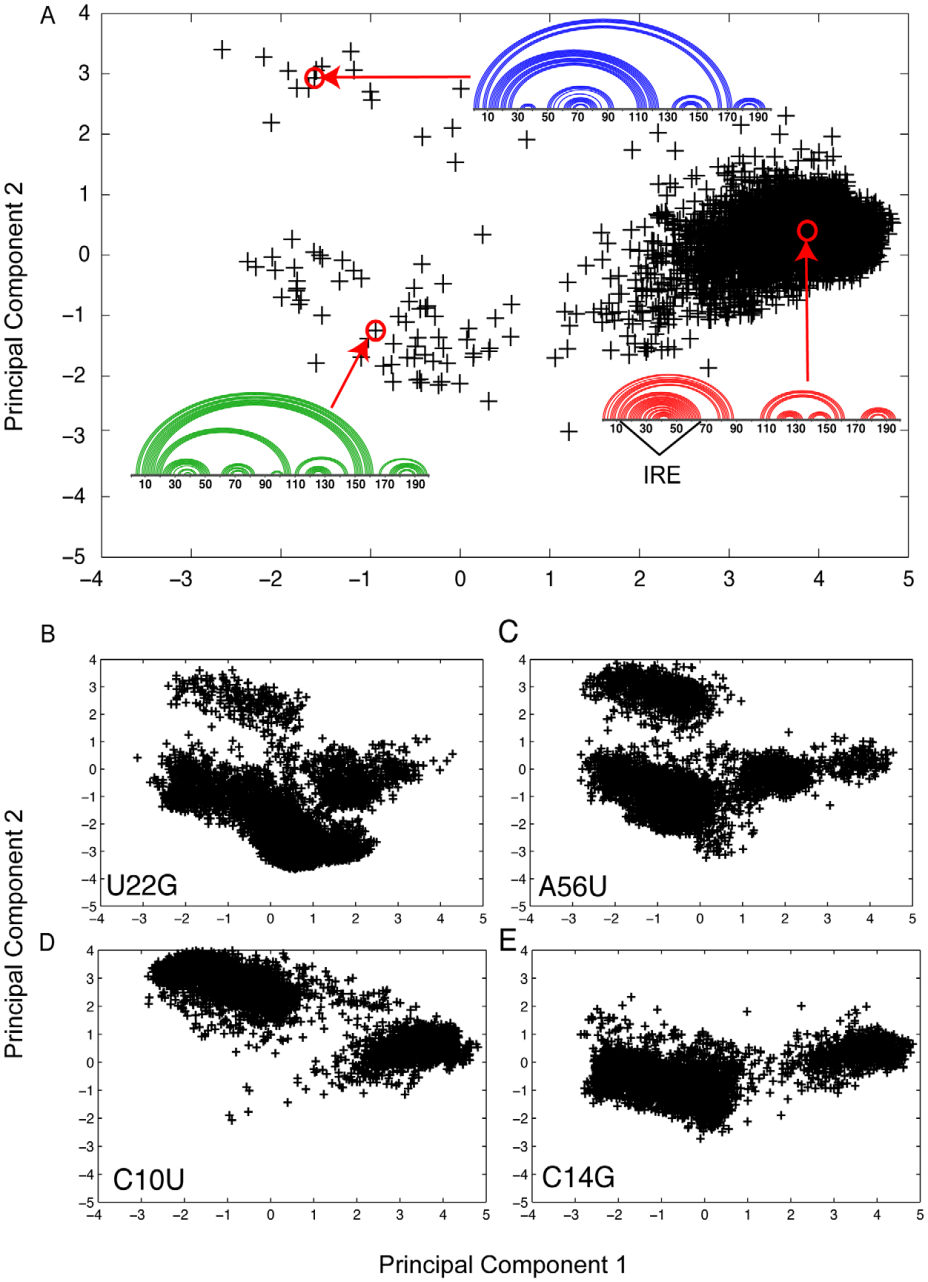
Structural analysis using Boltzmann sampling and principal cponent analysis of FTL 5′ UTR and four Hyperferritinemia cataract syndrome–associated mutations [**39**]. (A) Boltzmann sampling and principal component decomposition of 5000 alternative structures of the FTL 5′ non-diseased UTR. Each cross in the diagram represents one of the 5000 structures projected onto the first two principal components [40]. We use linear (or arc) diagrams to illustrate representative structures in the principal component space. In this case, three main clusters are observed, with the right, middle quadrant (red representative structure) being most highly populated for the WT sequence. Structures within this highly populated cluster all contain an IRE element (indicated in the figure), which has been shown to be critical in regulating FTL [39]. (B) Effect of the U22G mutation on the RNA structural ensemble involves populating both of the alternative RNA conformations. (C) A similar redistribution occurs with the A56U mutation. (D) Only the top, left hand cluster is populated with the disease-associated C10U mutation. (E) The C14G populated the lower, left hand quadrant, which also does not form the regulatory IRE.

One limitation of the partition function representation (Figure 1B, for example) is in the visualization and interpretation of the structural ensemble change induced by mutation. UTRs generally do not adopt single well-defined structures and classic representations of RNA structure (commonly referred to as “airport terminal diagrams”) cannot accurately be used to visualize overall changes in the ensemble. An alternative visualization of the structural ensemble is illustrated in Figure 3A for the wild-type (non-diseased) FTL 5′ UTR. We carried out a Boltzmann sampling of RNA structures using the sFold procedure [40], [41] and generated an ensemble of 5000 alternative RNA structures from the wild-type and mutant sequences. We then perform principal component analysis (PCA) on the full ensemble of structures. The ensemble of structures that belong to a particular sequence (wild-type or a specific mutant) were then projected onto the first two principle components as shown in Figure 3. This allows us to visualize the structural heterogeneity in the ensemble of structures for a sequence, keeping in mind that two points that are close together in our projection diagram indicate the two corresponding structures are similar in structural space.

For the FTL wild-type sequence we find that a majority of our sampled structures are grouped in a single cluster in the right center quadrant of the PCA graph. Representative structures for the three main structural clusters identified for FTL are illustrated in the Figure 3A insets as linear diagrams. We clearly see the formation of the IRE in the representative structure (red), indicating that a majority (97%) of wild-type RNAs adopt this structure. It is when we perform the same Boltzmann sampling procedure for the four diseased SNP sequences that we are able to visualize the nature of the structural ensemble change caused by these disease-associated mutations.

In Figure 3B–3E we project Boltzmann sampled structures onto the same principle components as those used in Figure 3A for the four Hyperferritinemia Cataract Syndrome associated SNP sequences. This analysis immediately reveals the nature of the structural change that putatively is the cause of the disease phenotype. The U22G and A56U mutations result in all three structural clusters populated (Figure 3B and 3C) while the C10U and C14G mutations selectively populate one of the mutant clusters (Figure 3D and 3E). In all cases, we find that the disease-associated mutations populate alternative conformations where the IRE is not formed. For FTL, the non-diseased UTR adopts a compact structural ensemble where the IRE is formed, while the diseased-associated SNPs shift the ensemble to include a significant number of structures where the IRE is disrupted in favor of long-range base pairs. In Table 2, we compute the relative population of the three clusters for the wild type and mutant sequences and find that all four disease-associated mutations significantly reduce the percentage of structures containing an IRE. Nonetheless, we see that no single mutation completely abolishes the cluster with the IRE, suggesting a shift in the relative populations of each conformation.

**Table 2:** Relative population of the three structural clusters for the FTL 5′ UTR.

	RED CLUSTER^1^	GREEN CLUSTER^2^	BLUE CLUSTER^3^
Wild-Type	98%	1.6%	0.4%
U22G	12%	83%	5%
A56U	17%	18%	64%
C10U	36%	0%	64%
C14G	25%	75%	0%

1 Middle-right quadrant in Figure 3, red structure containing IRE.

2 Lower-left quadrant in Figure 3, green structure.

3 Upper-left quadrant in Figure 3, blue structure.

### One phenotype, multiple genotypes

The four SNPs we identify in the 5′ UTR of FTL as having a large effect on its structural ensemble are a subset of the 30 SNPs associated with Hyperferritinemia Cataract Syndrome reported in HGMD. Since HGMD is based on existing published literature, one can assume that these 30 SNPs represent only a subset of all mutations that can cause the Hyperferritinemia phenotype. A majority (28) of the known SNPs associated with Hyperferritinemia Cataract Syndrome occur in the 5′ UTR of FTL, suggesting that the UTR is central in the regulation of the gene. The four mutations we identify using our partition function calculation and correlation analysis (which we will now refer to as the SNPFold algorithm) identify SNPs that have a major effect on the RNA structural ensemble. By design, SNPFold identifies the SNPs that alter the global structural ensemble of the RNA, and will not identify SNPs that have only local structural effects on the RNA. It is clear, however, that a global effect on the RNA structural ensemble is not a prerequisite for disease association. Clearly, multiple molecular mechanisms can cause the same phenotype; in the case of Hyperferritnaemia Cataract Syndrome any mutation that either directly or indirectly affects the IRE and its ability to bind the corresponding Iron Response Protein (IRP) can result in the phenotype.

In the supplement (Figure S2) we illustrate a natural extension of the SNPFold algorithm for analyzing multiple disease-associated SNPs. We average the change in base-pair probability for each nucleotide and for all Hyperferritinemia Cataract Syndrome associated SNPs. This global analysis of the effects of SNPs on the RNA structure clearly identifies the IRE in the 5′ UTR, which is where on average, the largest changes in base-pair probability are observed. As more associated genotypic information becomes available, it is likely that it will be possible to use this data to identify other RNA structural elements within the transcriptome.

## Discussion

Our analysis of the effects of disease-associated human genetic variation on mRNA and regulatory non-coding RNAs reveals the extent to which specific SNPs affect the RNA structural ensemble. The SNPfold algorithm we propose is unique in that it takes into account the effects of mutation on the ensemble of possible RNA structures, and not just a single minimum free energy structure. UTRs are not evolved to adopt a single, well-defined structure (unlike catalytic RNAs, for example [42]) but will rather adopt a large ensemble of structures [43]. We find that a majority of mutations have small, local effects on the structural ensemble (Figure 2), while certain specific mutations can profoundly alter it. In Figure S3, we compare the performance of MFE (mFold) algorithms to the partition function approach we used and show that our approach is far less sensitive to mutation. We identified those disease-associated mutations in human UTRs that have a large effect on the RNA structural ensemble and report them here.

We identified a broad range of disease phenotypes that are associated with SNPs that alter the RNA structural ensemble. For all the disease states presented in Table 1, the mRNA is either hypothesized or has been shown to play a causal role in the association. In certain cases, assays have already been carried out to show that the SNP causes a change in translation efficiency [26], [39], and/or mRNA stability [44], [45]. We also identified the mRNAs in which RIP-chip [46] experiments measured an interaction with an RNA binding protein (Table 1). We find that several RNA binding proteins including ELAVL1 (embryonic lethal, abnormal vision, Drosophila)-like 1), PABPC1 (Polyadenylate-binding protein 1), and IGFBP2 (insulin-like growth factor binding protein 2) are found to co-IP with our mRNAs of interest (Table 1 and Table S1). This suggests that the SNP induced structural changes could affect protein binding for the mRNAs identified in Table 1. Furthermore, our analysis of pre-mRNAs (Table S2) suggests that the conformational changes induced by SNPs are most significant in the mature mRNA. Finally, analysis of eQTL (expression Quantitative Trace Locus, Table S3) data reveals that for all but two of the common SNPs we identified in our RNA structural analysis, there is no measured effect on transcriptional levels [47].

To ascertain the relationship between our predicted changes in base-pairing probability and RNA functional elements we performed additional analyses reported in the supplement (Figure S4). We find that predicted changes in base-pairing probability overlap significantly with known RNA functional elements including IREs, IRES (Internal Ribosome Entry Sites), uORFs (upstream Open Reading Frames), PAS's (Polyadenylation Sites), TOPs (Terminal Oligopyrimidine tracts), MBEs (Musashi Binding Elements), K-Boxes and GY-Boxes. The IRES is an alternate translation initiation site that allows the ribosome to bind the mRNA in a 5′ cap independent manner [48]. uORFs are found upstream of the normal ORF and lower the translation of the main ORF, and in some cases lead to the production of a short regulatory transcript [49], [50]. A PAS is a variable AU-rich sequence that is essential for the recruitment of the polyadenylation machinery needed to add the polyA tail to a given RNA [51]. TOP elements tag the mRNA for growth associated translational repression [52]. MBEs recruit and bind the Musashi protein, an evolutionarily conserved RNA-binding protein known to have the ability to regulate mRNA translation [53]. K-Boxes and GY-Boxes are conserved negative regulators, acting as binding platforms for the 5′ seed regions of miRNAs [54], [55]. We therefore observe SNP induced changes in base-pairing probability in a majority of the RNA functional elements in our UTRs of interest. For each of these elements, accessibility is key to function, and the base-pairing probability changes we predict affect accessibility.

We performed a complete analysis of the structural changes caused by disease associated mutations in the 5′ UTR of FTL, because it is already established that an IRE is present in the UTR and is responsible for regulating FTL [26], [39]. Our structural analysis of the FTL 5′ UTR (Figure 3) begins to reveal the molecular complexity of disease caused by mRNA structural rearrangement. We see in Figure 3 that no single SNP has the exact same effect on the structural ensemble. Nonetheless, the structural changes observed are limited in the case of this phenotype to three major structural clusters. Mutations shift the equilibrium between the different structural clusters. However, all structures sampled when projected in principal component space fall into these same clusters. A different behavior is observed in the 5′ UTR of RB1 (retinoblastoma 1), where the two disease-associated SNPs we identified also significantly repartition the structural ensemble (Figure S1). In this case, the disease-associated SNPs have the opposite effect to that observed in the FTL 5′ UTR. For the RB1 5′ UTR, the Retinoblastoma associated SNPs collapse the structural ensemble from three clusters to one.

Structural rearrangement of a UTR as a post-transcriptional regulatory mechanism is common in bacterial Riboswitches [16], [20]. In this case, the binding of a small molecule, in general a metabolite, changes the secondary structure of the RNA so as to promote or inhibit Ribosomal binding and gene translation [16]. It is therefore not surprising that certain specific mutations can have profound structural consequences on a human UTR. The UTRs and their associated SNPs we report here are in fact a type of “RiboSNitch,” that is a molecular switch that is activated by SNP. Unlike the Riboswitch, however, a RiboSNitch results in a permanent change in regulation and thus leads to the disease phenotype. RiboSNitches represent a novel therapeutic target, since small molecules can repartition the RNA structural ensemble.

The U310A and U336A mutations in the 5′UTR of CPB2 are particularly noteworthy. CPB2 codes for the Thrombin-Activable Fibrinolysis Inhibitor (TAFI) [45]. An activated form of TAFI is known to slow down Fibrinolysis [44]. Mutations that alter the expression level of this protein are associated with various thrombotic disorders, including ischemic stroke [56]. Results from mRNA decay assays show the presence of these SNPs result in an mRNA with an altered stability [45]. Our results suggest that the associated SNPs significantly alter the RNA conformational ensemble of the TAFI 5′ UTR and that this could affect RNA decay. Therefore, conformational change is also a likely determinant of mRNA stability which indirectly controls protein expression.

Low-cost whole genome sequencing, SNP microarrays specifically focused on non-coding regions of the genome, and greater phenotypic information available through electronic medical records will necessarily yield new phenotypic associations in the non-coding regions of the genome. The SNPfold algorithm provides a novel approach to gain structural insight into the structural consequences of mutations on a transcript. We therefore developed a web server (http://cloud.wadsworth.org/snpfold) that reproduces the computational functionality we describe in this manuscript. In particular our web server allows the simultaneous analysis of multiple SNPs. This computational tool will provide the GWAS community with a simple way to quantitatively evaluate the effects of SNPs (and other mutations) on the RNA structural ensemble.

## Materials and Methods

### Identification of a set of disease-associated SNPs in UTRs

The Human Genetic Mutations Database (http://www.hgmd.cf.ac.uk/) was utilized [35], [36] as a primary source of genotype/phenotype associations in our study. The professional version of the database, obtainable through a yearly subscription fee, contains the “prom” table. The 2009.1 version of HGMD that we utilized contains 1459 entries in the prom table. Each entry contains DNA sequences that flank the disease associated SNP. These flanking sequences were mapped to the human reference genome, in order to determine the genomic coordinates of the corresponding SNPs [37]. 1385 mutations from this table were successfully mapped to some specific coordinate within a specified chromosome.

Once the coordinates of the SNPs in the table were obtained, the ‘refgene’ table from the hg18 build of the Human genome [38] was used to identify SNPs that map on a UTR of a gene. For a given gene transcript, the corresponding chromosome and strand are provided, as well as coordinates of the transcription and translation start/stop sites, and the exon start/stop sites. SNPs whose coordinates map between the transcription start/translation stop sites or the translation stop/transcription stop sites were classified as mapping onto a UTR region. SNPs that either mapped onto intronic regions of UTRs (not between an exon start and stop coordinate) or were less than 10 nucleotides away from either end of the UTR were excluded from our analysis.

### Obtaining sequences of UTR regions

The gene coordinates in ‘refgene’ were used to extract UTR sequences for a given disease associated UTR SNP in ‘prom’. For this, full sequences for each chromosome in the human reference genome were required. We used UCSC genome build hg18 [37]. If the gene was on the ‘minus’ strand, we used the reverse complement of the extracted sequence, as the human reference genome consists entirely of sequence from the ‘plus’ strand. Using the mapped coordinates for each UTR SNP, two different UTR sequences were produced: the wild type sequence, and the sequence containing the disease-associated SNP. It should also be noted that the UTR sequences produced were from the mature transcripts, and are fully spliced.

### SNPfold algorithm

The SNPfold algorithm that was developed utilizes the RNA partition function calculations implemented in RNAfold [57], [58]. The algorithm requires an input of two different RNA strands that are identical in length. For the analysis of any RNA SNP, the wild type RNA sequence and the RNA sequence containing the disease associated SNP of interest was obtained as previously described. The sum of the columns of each partition function was used to compute the Pearson Correlation coefficient for each WT/SNP pair.

To normalize for sequence length, we computed a non-parametric p-value for a given correlation coefficient. This value represents the likelihood of a random mutation in the RNA of interest producing the same or lower correlation coefficient. For a sequence of length *n* all possible *3n* mutations are computed and the mutation of interest ranked compared to all the other possible mutations. The non-parametric p-value was then estimated as the rank of the mutation of interest divided by *3n*.

### Principal Component analysis of the structural ensemble

The structures for the Principal Component analysis were generated using the statistical sampling algorithm in the sFold software [40]. The structures were then parameterized into a vector of ones and zeros (with one representing the base being paired). A sample of 1000 structures from each mutant and WT sequence was randomly selected and used to generate the basis vectors of the principle component analysis. The two firsty basis vectors representing the variances in the data were used to project the 5000 structures from each sequence onto the same principle components. The resultant data took the form of a 2D scatterplot. The linear structure diagrams for the wild type were generated using the VARNA software [59].

### Scanning UTRs for RNA regulatory motifs

A search for known RNA regulatory motifs was carried out in every UTR reported in Table 1 and Table S1. The UTRscan algorithm (which searches a user-submitted RNA sequence for known UTR motifs listed in the UTRsite database) was utilized [60], [61]. In 3′ UTRs, an additional search for miRNA binding sites was conducted using RegRNA which predicts splicing sites and miRNA binding sites in mRNA sequences [62].

### Detection of RBP binding to transcripts of interest

RIP-Chip Data obtained from Scott Tenenbaum (UAlbany) was analyzed in the context of the mRNAs reported in Table 1 and Table S1 [46]. The data included analyses of RNA transcript coprecipitation with three different RNA-binding proteins (Elavl1, Pabpc1, and Igfbp2) in two different cell lines (Gm12878 and K562). p-values (−log10) above 1.3 were deemed statistically significant for RNA binding, and are reported in Table 1 and Table S1.

### LD and eQTL analysis of SNPs

We searched dbSNP to identify common variants (SNPs) with accession IDs (rs numbers) from Table 1 and Table S1. For the mRNAs in which we identified common variants, LD data from HapMap was downloaded [63] and reported above a significant (R^2^>0.9) threshold. eQTL data from [64] was queried using the common dbSNP IDs.

## Supporting Information

Figure S1 Principal component decomposition of Boltzmann sampling of the RB1 5′ UTR where mutations are found to be associated with Retinoblastoma [65]. (A) Wild-type structural sampling showing four distinct clusters; representative structures for each cluster are presented as blue arc diagrams. The three upper clusters are most populated, with 98% of the structures. (B) Effects of the disease-associated G17C mutation on the RNA structural ensemble. The mutation causes a radical shift towards an alternative structure with far fewer long-range interactions. (C) Effects of G18U on the structural ensemble resulting in a complete shift in structures as well.
(0.97 MB PDF)

Figure S2 Average change in base-pair probability due to mutation for the 30 known Hyperferritinaemia Cataract Syndrome associated SNPs. SNP locations are indicated as vertical green lines, and the average change is plotted in red. This graph clearly identifies the largest average changes in nucleotides 20–50, which make up an Iron Response Element in the 5′ UTR of the FTL mRNA.
(0.60 MB PDF)

Figure S3 Comparison of WT/SNP correlation coefficient distributions for all possible mutations in nine selected UTRs in which we have identified a putative RiboSNitch (see Table 1). The black line is using our novel partition function calculation, while the red line is using a standard minimum free energy (MFE) approach (like mFold). The partition function calculation is far less sensitive to mutations and produces a continuously decreasing distribution, allowing us to accurately estimate the significance of a conformational change and will thus lead to fewer false-positives.
(0.29 MB PDF)

Figure S4 Schematic representations (heat maps) of the change in base-pairing probability upon disease-associated SNP mutations in their respective UTRs. Red indicates high differences in base-pairing probability between the wild-type and disease genotype. Motifs detected using the UTRscan program are indicated with green boxes. miRNA binding targets in 3′UTRs detected with RegRNA are indicated via blue boxes. Gene names, 5′ or 3′ UTR and UTR length are indicated under each diagram, and the corresponding SNP is indicated to the left of each heatmap.
(0.70 MB PDF)

Figure S5 pre-mRNA gene maps of SNPs that are in high LD (R2>0.9) with our predicted RiboSNitch SNPs. Exonic regions are indicated as thick lines, introns as thin horizontal lines. Vertical black lines indicate the postions of high LD SNPs. SNPs that cause missense mutations in the coding region of the listed gene are colored in pink, and have an associated rs number listed above their respective positon. (A) rs1087 (in CPB2 3′UTR, 427 nt), (B) rs1087 (in CPB 3′UTR, 453 nt), (C) rs8004738 (in SERPINA1 5′UTR, 533 nt), (D) rs8004738 (in SERPINA1 5′UTR, 551 nt), (E) rs8004738 (in SERPINA1 5′UTR, 551 nt), (F) rs5051 (in AGT 5′UTR, 508 nt), (G) rs5050 (in AGT 5′UTR, 508 nt), (H) rs1010167 (in GSTM4 5′UTR, 314 nt), (I) rs1799794 (in XRCC3 5′UTR, 380 nt), (J) rs6141 (in THPO 3′UTR, 528 nt), (K) rs2016520 (in PPARD 5′UTR, 309 nt), (L) rs2302009 (in CCL26 3′UTR, 169 nt), (M) rs12386703 (in PEX1 5′UTR, 96 nt).
(0.39 MB PDF)

Table S1 Disease states and phenotypes in which one associated SNP was found to alter the structural ensemble of the RNA.
(0.09 MB PDF)

Table S2 (A) Lengths of pre- and mature mRNAs where we have identified a RiboSNitch. (B) SNPFold analysis of RiboSNitch in pre and mature mRNA UTRs revealing that a majority of the RiboSNitches identified affect the mature mRNA only. The * indicates an approximate p-value computed from a distribution of random sequences, due to the computational limitations of calculating the p-value for the longer (>2000) pre-mRNAs.
(0.40 MB PDF)

Table S3 eQTL data for common variant SNPs identified as potential RiboSNitches. A minority of the SNPs we identified affect transcriptional levels.
(0.06 MB PDF)
