# Supplementary figures and images for: Disease-Associated Mutations That Alter the RNA Structural Ensemble
Source: PLoS Genet. 2010 Aug 19;6(8):e1001074. doi: 10.1371/journal.pgen.1001074 (PMC2924325; doi:10.1371/journal.pgen.1001074)

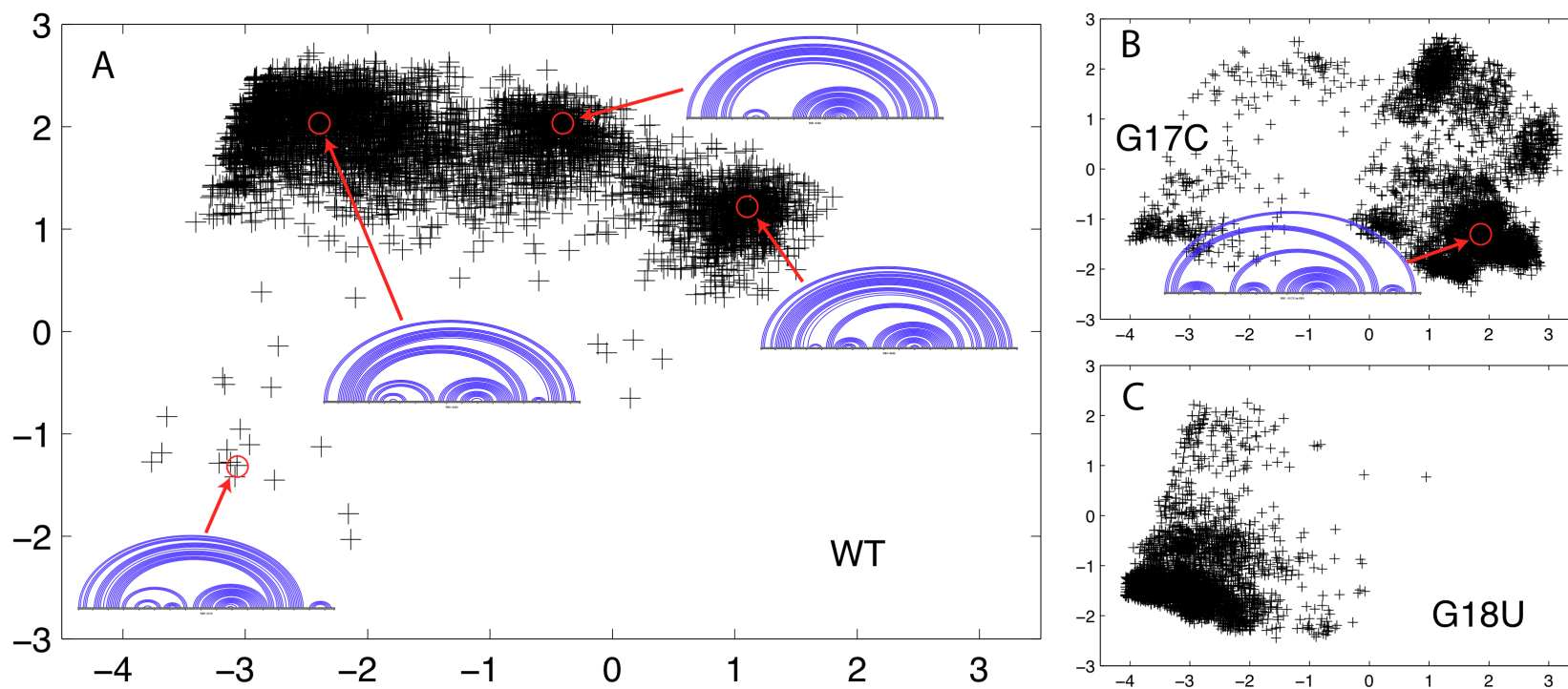

Figure S1

Supplement: Figure S1 — Principal component decomposition of Boltzmann sampling of the RB1 5′ UTR where mutations are found to be associated with Retinoblastoma [65]. (A) Wild-type structural sampling showing four distinct clusters; representative structures for each cluster are presented as blue arc diagrams. The three upper clusters are most populated, with 98% of the structures. (B) Effects of the disease-associated G17C mutation on the RNA structural ensemble. The mutation causes a radical shift towards an alternative structure with far fewer long-range interactions. (C) Effects of G18U on the structural ensemble resulting in a complete shift in structures as well. (0.97 MB PDF) [file pgen.1001074.s001.pdf]

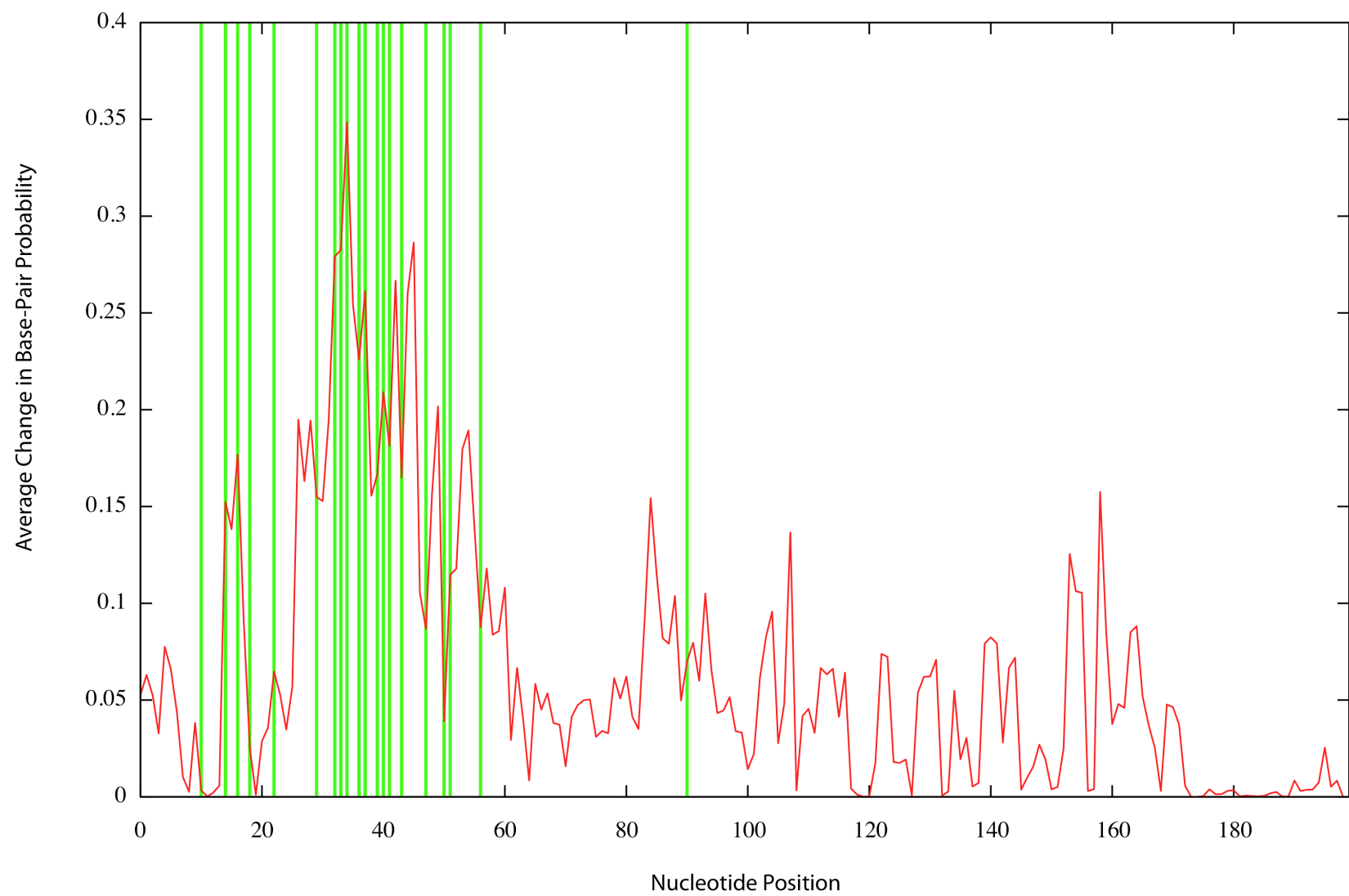

Figure S2

Supplement: Figure S2 — Average change in base-pair probability due to mutation for the 30 known Hyperferritinaemia Cataract Syndrome associated SNPs. SNP locations are indicated as vertical green lines, and the average change is plotted in red. This graph clearly identifies the largest average changes in nucleotides 20–50, which make up an Iron Response Element in the 5′ UTR of the FTL mRNA. (0.60 MB PDF) [file pgen.1001074.s002.pdf]

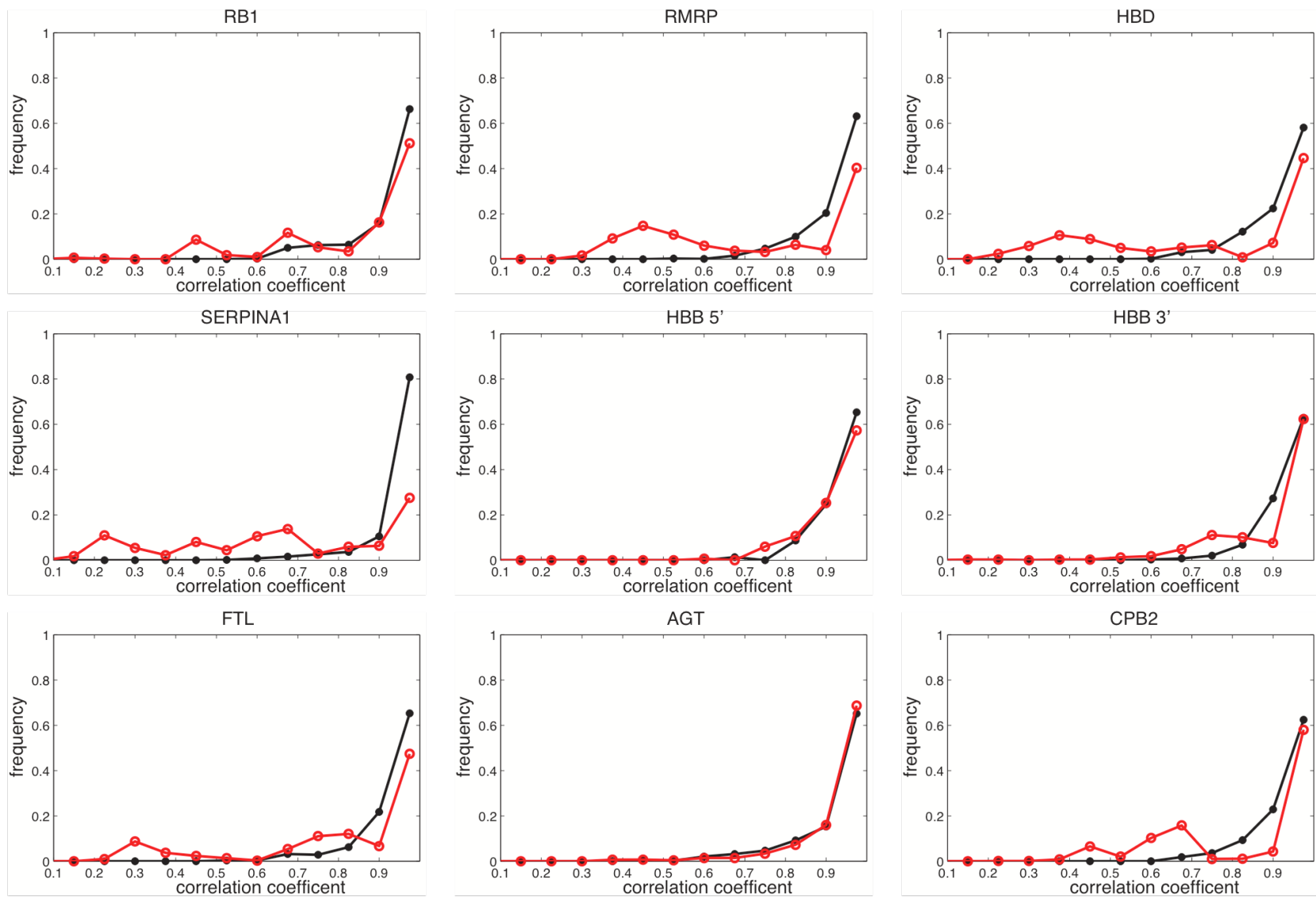

Figure S3

Supplement: Figure S3 — Comparison of WT/SNP correlation coefficient distributions for all possible mutations in nine selected UTRs in which we have identified a putative RiboSNitch (see Table 1). The black line is using our novel partition function calculation, while the red line is using a standard minimum free energy (MFE) approach (like mFold). The partition function calculation is far less sensitive to mutations and produces a continuously decreasing distribution, allowing us to accurately estimate the significance of a conformational change and will thus lead to fewer false-positives. (0.29 MB PDF) [file pgen.1001074.s003.pdf]

A)

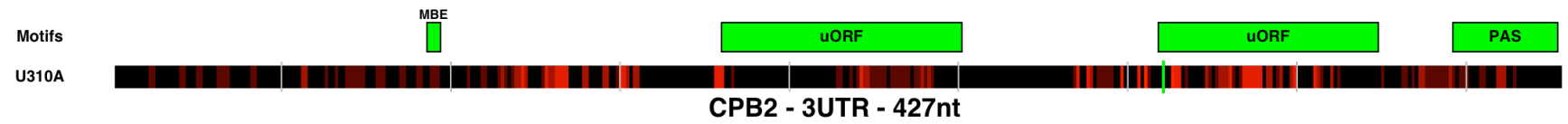

B)

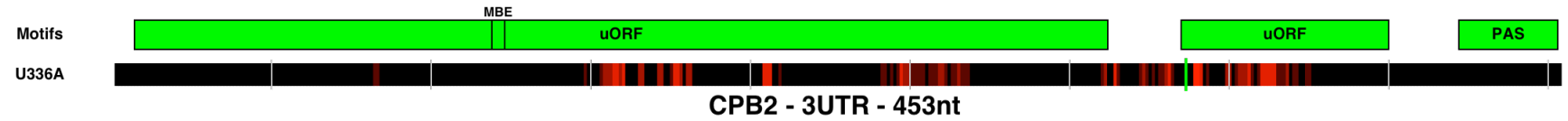

C)

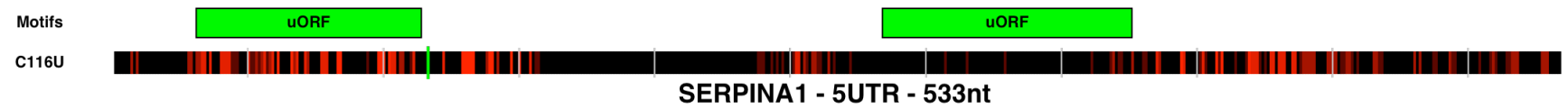

D)

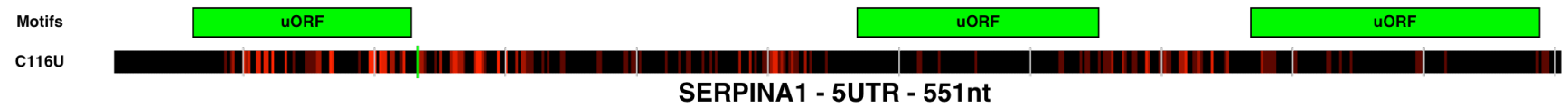

E)

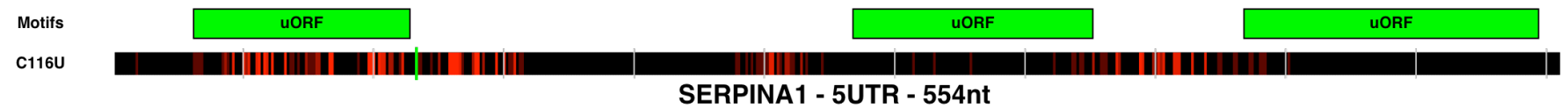

F)

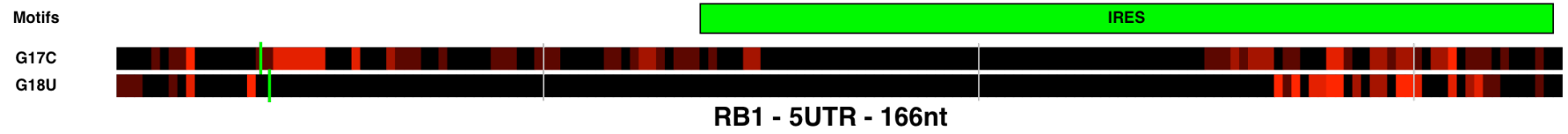

G)

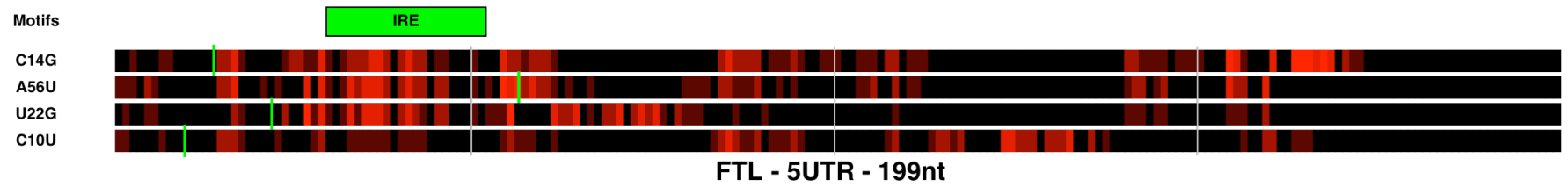

H)

miR Binding Sites

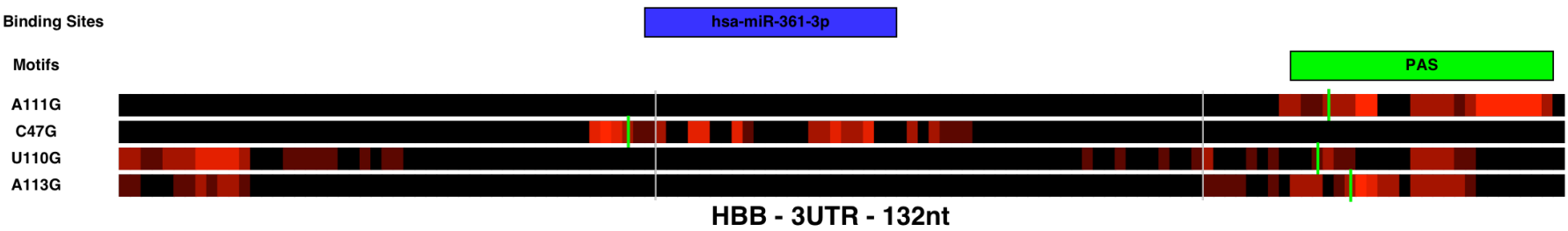

I)

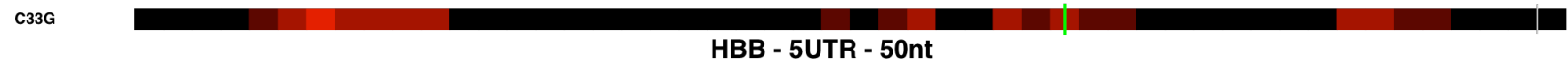

J)

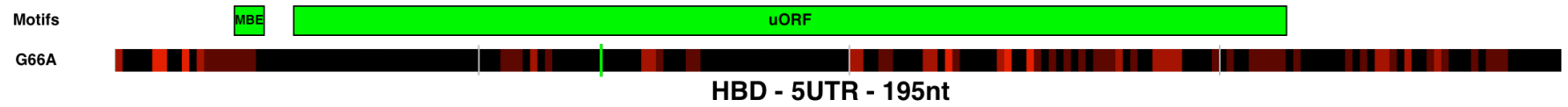

K)

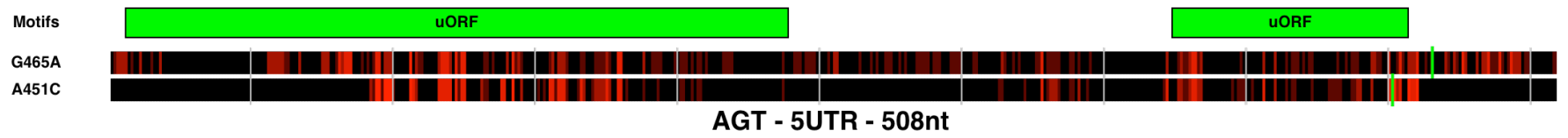

L)

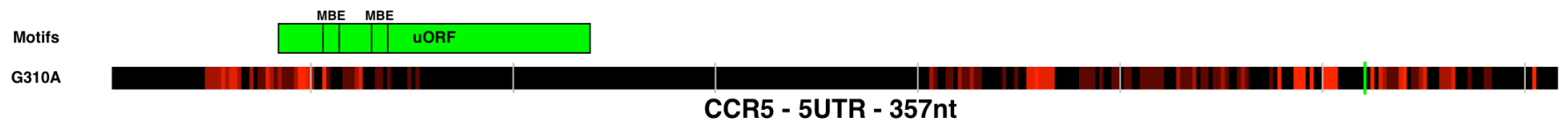

M)

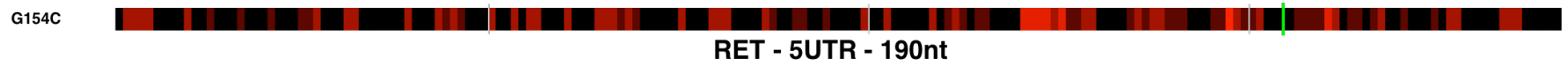

N)

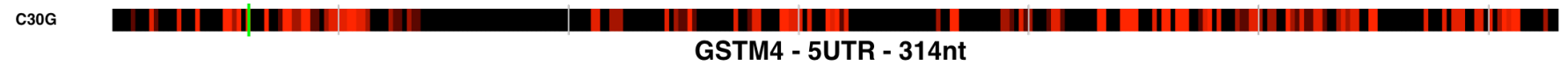

O)

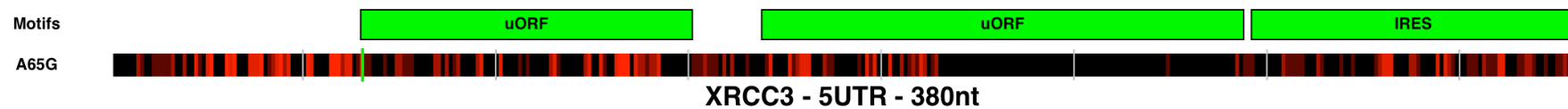

P)

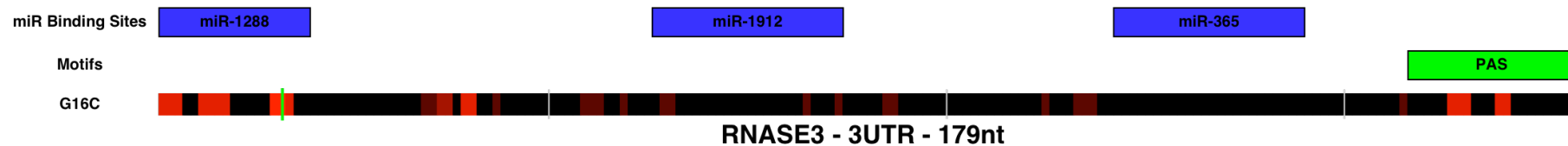

Q)

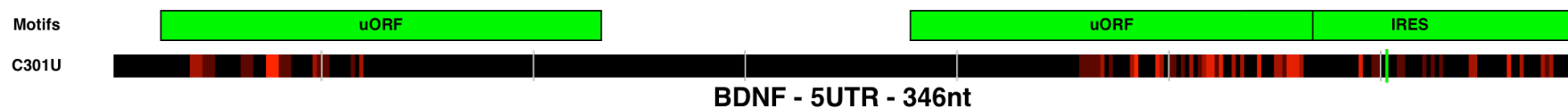

R)

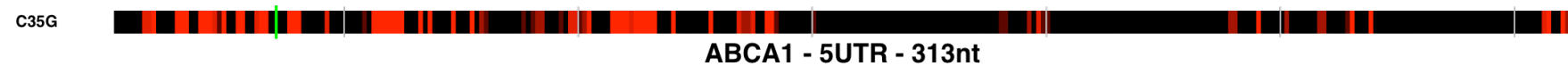

S)

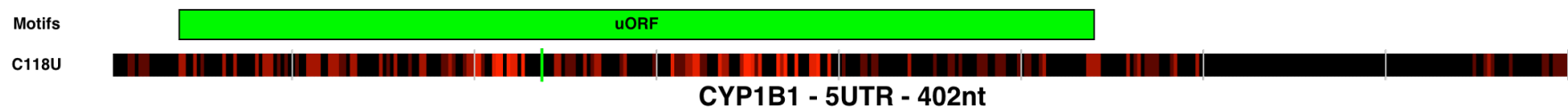

T)

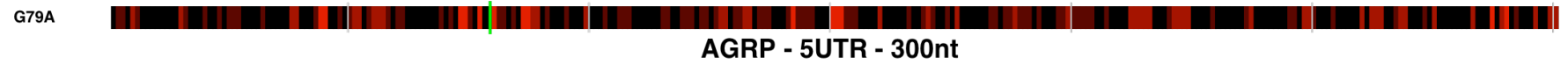

U)

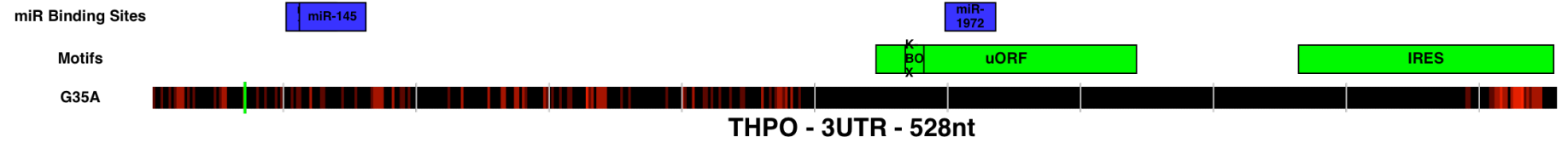

V)

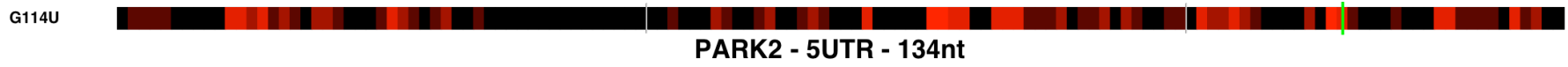

W)

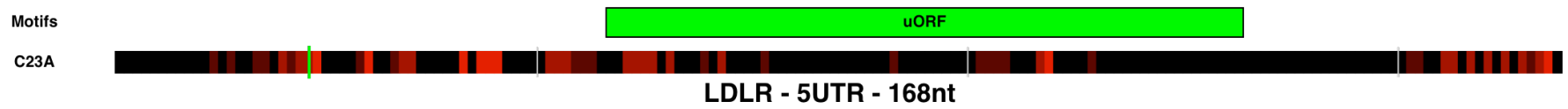

X)

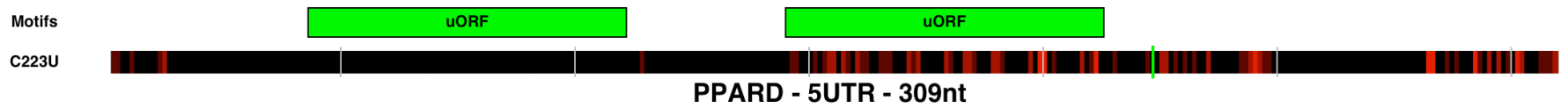

Y)

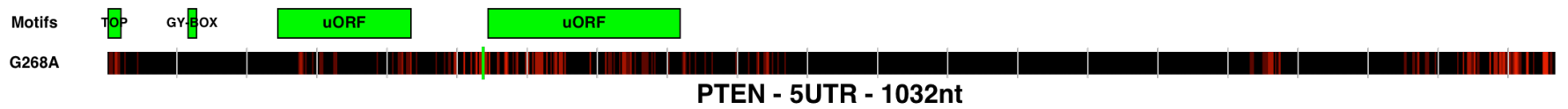

Z)

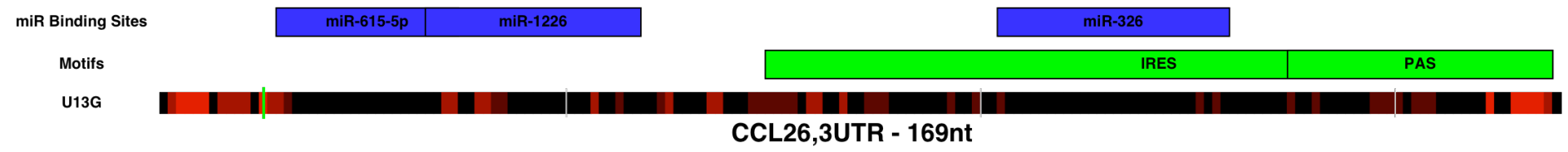

AA)

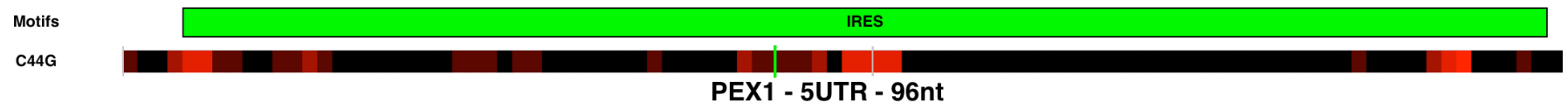

Figure S4

Supplement: Figure S4 — Schematic representations (heat maps) of the change in base-pairing probability upon disease-associated SNP mutations in their respective UTRs. Red indicates high differences in base-pairing probability between the wild-type and disease genotype. Motifs detected using the UTRscan program are indicated with green boxes. miRNA binding targets in 3′UTRs detected with RegRNA are indicated via blue boxes. Gene names, 5′ or 3′ UTR and UTR length are indicated under each diagram, and the corresponding SNP is indicated to the left of each heatmap. (0.70 MB PDF) [file pgen.1001074.s004.pdf]

A)

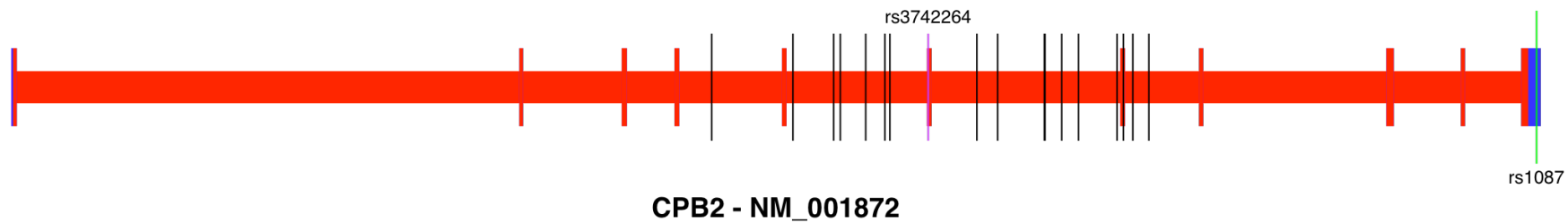

B)

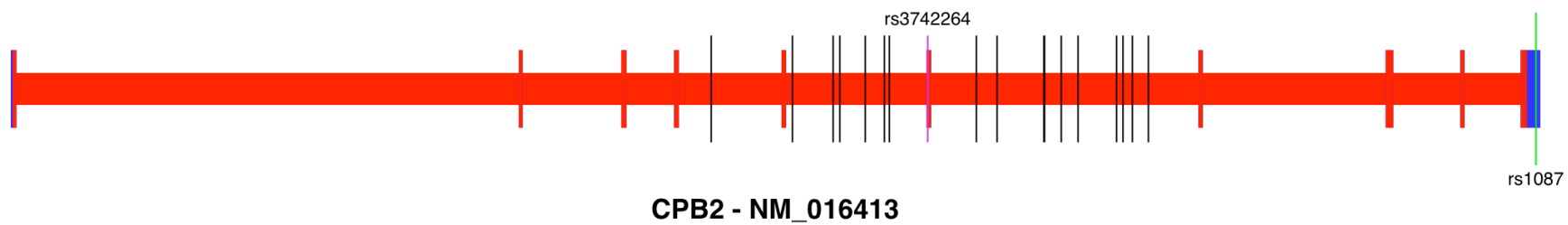

C)

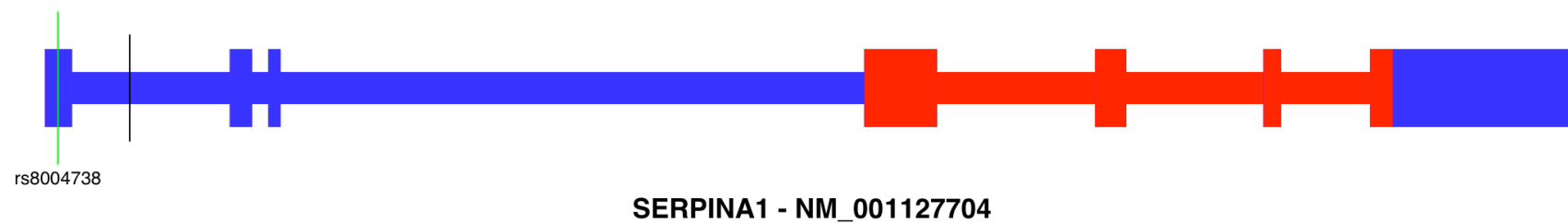

D)

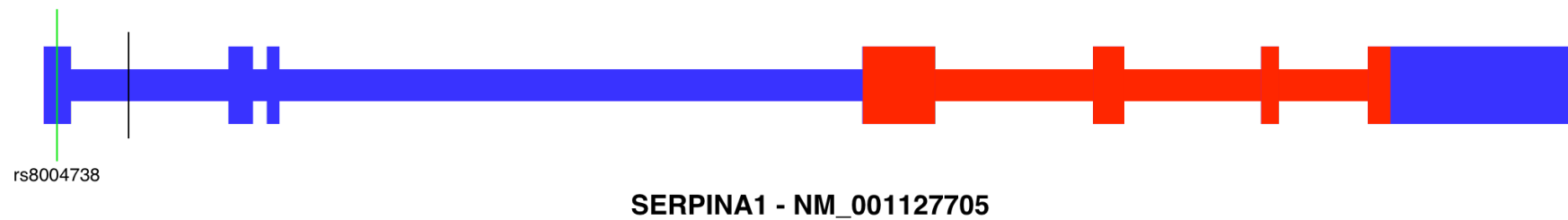

E)

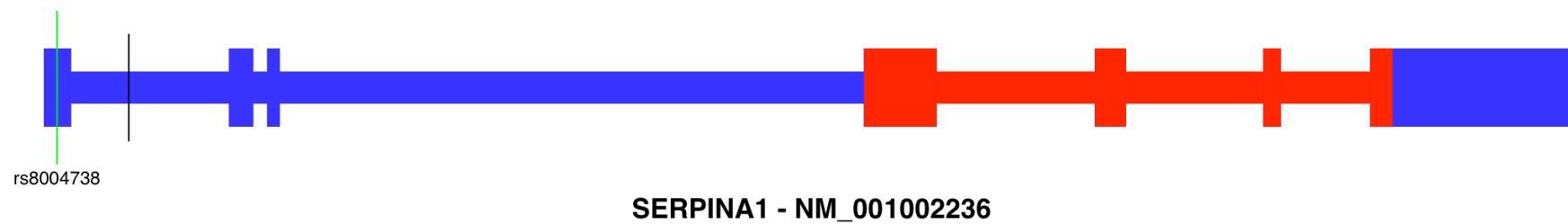

F)

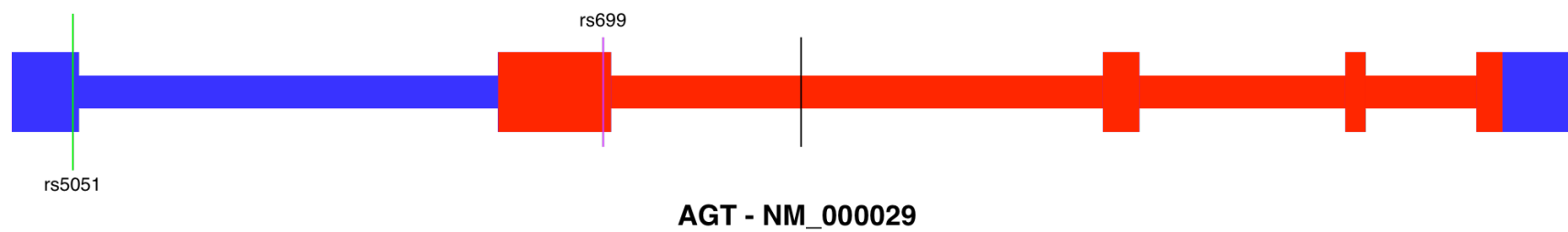

G)

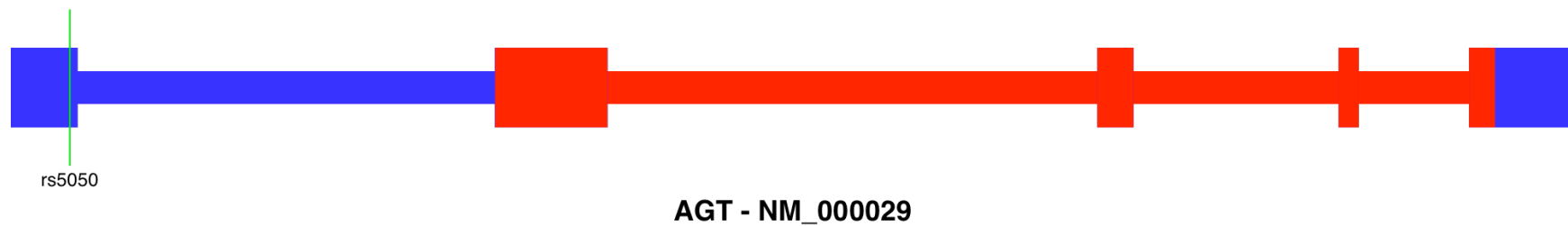

H)

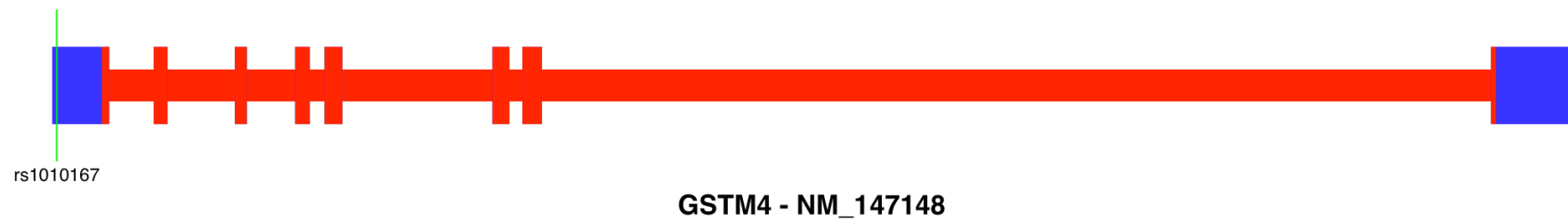

I)

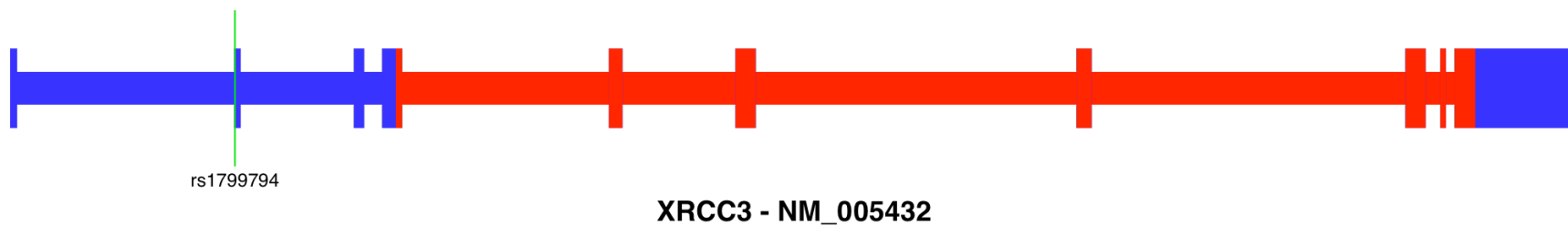

J)

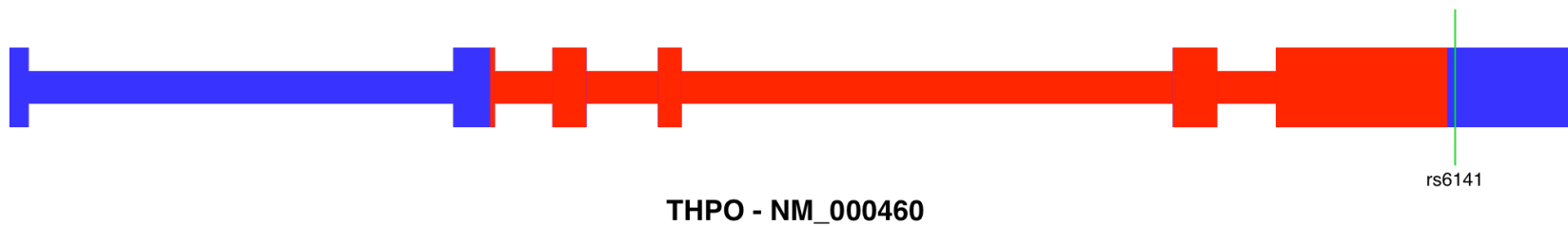

K)

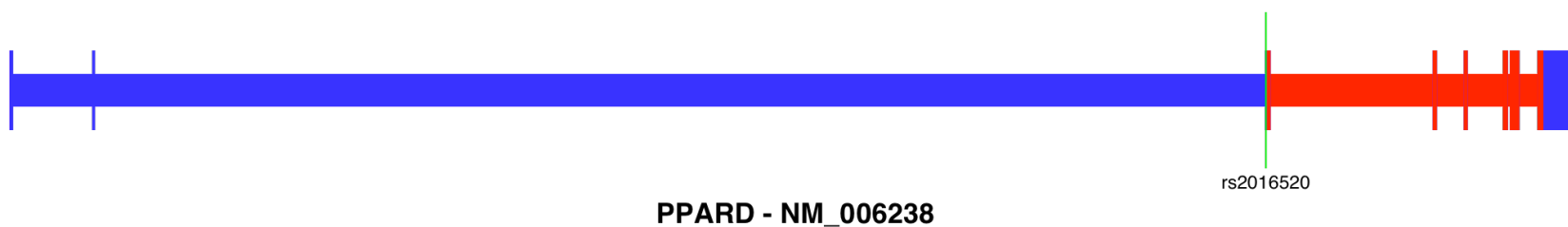

L)

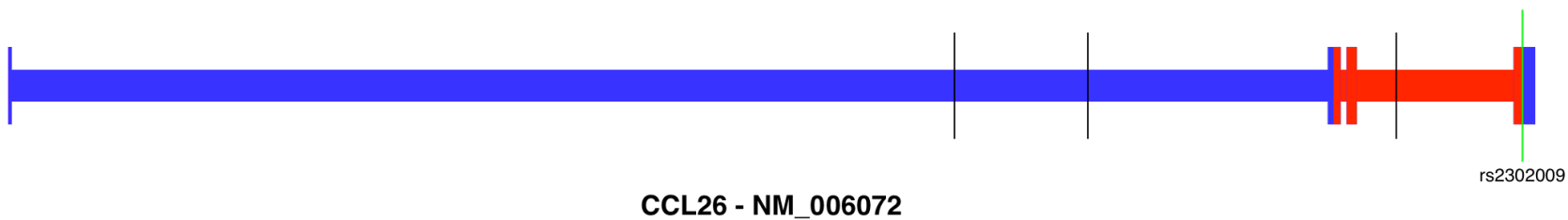

M)

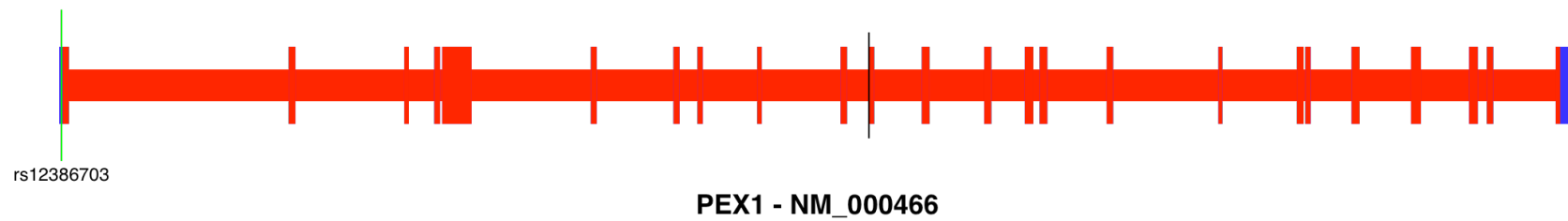

Figure S5

Supplement: Figure S5 — pre-mRNA gene maps of SNPs that are in high LD (R2>0.9) with our predicted RiboSNitch SNPs. Exonic regions are indicated as thick lines, introns as thin horizontal lines. Vertical black lines indicate the postions of high LD SNPs. SNPs that cause missense mutations in the coding region of the listed gene are colored in pink, and have an associated rs number listed above their respective positon. (A) rs1087 (in CPB2 3′UTR, 427 nt), (B) rs1087 (in CPB 3′UTR, 453 nt), (C) rs8004738 (in SERPINA1 5′UTR, 533 nt), (D) rs8004738 (in SERPINA1 5′UTR, 551 nt), (E) rs8004738 (in SERPINA1 5′UTR, 551 nt), (F) rs5051 (in AGT 5′UTR, 508 nt), (G) rs5050 (in AGT 5′UTR, 508 nt), (H) rs1010167 (in GSTM4 5′UTR, 314 nt), (I) rs1799794 (in XRCC3 5′UTR, 380 nt), (J) rs6141 (in THPO 3′UTR, 528 nt), (K) rs2016520 (in PPARD 5′UTR, 309 nt), (L) rs2302009 (in CCL26 3′UTR, 169 nt), (M) rs12386703 (in PEX1 5′UTR, 96 nt). (0.39 MB PDF) [file pgen.1001074.s005.pdf]
