# Supplementary material for: Disease-Associated Mutations That Alter the RNA Structural Ensemble
Source: PLoS Genet. 2010 Aug 19;6(8):e1001074. doi: 10.1371/journal.pgen.1001074 (PMC2924325; doi:10.1371/journal.pgen.1001074)
Supplement: Table S1 — Disease states and phenotypes in which one associated SNP was found to alter the structural ensemble of the RNA. (0.09 MB PDF) [file pgen.1001074.s006.pdf]

Table S1: Disease states and phenotypes in which one associated SNP was found to alter the structural ensemble of the RNA.

Disease	Gene	HGMD Acc. #	UTR	UTR length	mutation	Corr. Coeff	P-value	pmid	reference	Motifs[1]	RBP[2] Binding	dbSNP[3] Ref. ID
Loss of protection against HIV infection	CCR5	CR084787	5	357	G310A	0.665	0.005	18094161	[1]	uORF, MBE 2X	-	-
Hirschprung Disease	RET	CR033995	5	190	G154C	0.617	0.018	14633923	[2]	-	ELAVL1, PABPC1	-
Change in Detoxification ability	GSTM4	CR040571	5	314	C30G	0.406	0.018	15128050	[3]	-	PABPC1, IGFBP2	rs1010167*
Epith. ovarian cancer, serous type, prot., assoc.	XRCC3	CR057423	5	380	A65G	0.601	0.022	15924337	[4]	IRES, uORF 2X	-	rs1799794*
Allergy, assoc. with	RNASE3	CR067512	3	179	G16C	0.789	0.024	16434694	[5]	PAS	-	rs2233860
Alzheimer disease, association with	BDNF	CR014434	5	346	C301T	0.851	0.035	11244490	[6]	IRES, uORF 2X	ELAVL1, PABPC1	rs56164415
Increased triglyceride levels, association with	ABCA1	CR025352	5	313	C35G	0.566	0.036	11940086	[7]	-	ELAVL1, PABPC1	-
Glaucoma, primary congenital	CYP1B1	CR032431	5	402	C118T	0.675	0.037	12598442	[8]	uORF	-	-
Reduced promoter activity, association with	AGRP	CR073538	5	300	G79A	0.585	0.052	17180153	[9]	-	ELAVL1	rs34018897
Myocardial infarction, association with	THPO	CR014438	3	528	G35A	0.867	0.053	11257273	[10]	IRES, K-BOX, uORF	-	rs6141*
Parkinson disease, autosomal recessive	PARK2	CR024270	5	134	G114T	0.504	0.067	11971093	[11]	-	ELAVL1	-
Hypercholesterolaemia	LDLR	CR973644	5	168	C23A	0.745	0.083	9259195	[12]	uORF	-	-
Increased LDL cholesterol, association with	PPARD	CR035869	5	309	C223T	0.804	0.085	12615676	[13]	uORF 2X	ELAVL1	rs2016520*
Cowden's disease	PTEN	CR032089	5	1032	G268A	0.867	0.087	12844284	[14]	uORF,GYBOX,TOP	-	-
Eosinophilic oesophagitis, association with	CCL26	CR066323	3	169	T13G	0.679	0.097	16453027	[15]	IRES, PAS	ELAVL1, PABPC1	rs2302009*
Alleviation of zellweger syndrome	PEX1	CR053503	5	96	C44G	0.818	0.097	16088892	[16]	IRES	ELAVL1	rs12386703*

[1]Structural and sequence motifs identified in mRNA UTRs using UTRScan[17].
[2]RNA Binding Protein as determined by RIP-chip[18].
[3]dbSNP reference IDs for common variants. A star (*) indicates LD data is available and reported in Supplementary Figure S6.

1. Jin Q, Agrawal L, Meyer L, Tubiana R, Theodorou I, et al. (2008) CCR5Delta32 59537-G/A promoter polymorphism is associated with low translational efficiency and the loss of CCR5Delta32 protective effects. J Virol 82: 2418-2426.
2. Garcia-Barcelo M, Sham MH, Lee WS, Lui VC, Chen BL, et al. (2004) Highly recurrent RET mutations and novel mutations in genes of the receptor tyrosine kinase and endothelin receptor B pathways in Chinese patients with sporadic Hirschsprung disease. Clin Chem 50: 93-100.
3. Guy CA, Hoogendoorn B, Smith SK, Coleman S, O'Donovan MC, et al. (2004) Promoter polymorphisms in glutathione-S-transferase genes affect transcription. Pharmacogenetics 14: 45-51.
4. Auranen A, Song H, Waterfall C, Dicioccio RA, Kuschel B, et al. (2005) Polymorphisms in DNA repair genes and epithelial ovarian cancer risk. Int J Cancer 117: 611-618.
5. Jonsson UB, Bystrom J, Stalenheim G, Venge P (2006) A (G->C) transversion in the 3' UTR of the human ECP (eosinophil cationic protein) gene correlates to the cellular content of ECP. J Leukoc Biol 79: 846-851.
6. Kunugi H, Ueki A, Otsuka M, Isse K, Hirasawa H, et al. (2001) A novel polymorphism of the brain-derived neurotrophic factor (BDNF) gene associated with late-onset Alzheimer's disease. Mol Psychiatry 6: 83-86.
7. Zwarts KY, Clee SM, Zwinderman AH, Engert JC, Singaraja R, et al. (2002) ABCA1 regulatory variants influence coronary artery disease independent of effects on plasma lipid levels. Clin Genet 61: 115-125.
8. Ohtake Y, Tanino T, Suzuki Y, Miyata H, Taomoto M, et al. (2003) Phenotype of cytochrome P4501B1 gene (CYP1B1) mutations in Japanese patients with primary congenital glaucoma. Br J Ophthalmol 87: 302-304.
9. Sozen MA, de Jonge LH, Greenway F, Ravussin E, Smith SR, et al. (2007) A rare mutation in AgRP, +79G>A, affects promoter activity. Eur J Clin Nutr 61: 809-812.
10. Webb KE, Martin JF, Hamsten A, Eriksson P, Iacoviello L, et al. (2001) Polymorphisms in the thrombopoietin gene are associated with risk of myocardial infarction at a young age. Atherosclerosis 154: 703-711.
11. Hedrich K, Marder K, Harris J, Kann M, Lynch T, et al. (2002) Evaluation of 50 probands with early-onset Parkinson's disease for Parkin mutations. Neurology 58: 1239-1246.
12. Day IN, Whittall RA, O'Dell SD, Haddad L, Bolla MK, et al. (1997) Spectrum of LDL receptor gene mutations in heterozygous familial hypercholesterolemia. Hum Mutat 10: 116-127.
13. Skogsberg J, Kannisto K, Cassel TN, Hamsten A, Eriksson P, et al. (2003) Evidence that peroxisome proliferator-activated receptor delta influences cholesterol metabolism in men. Arterioscler Thromb Vasc Biol 23: 637-643.
14. Zhou XP, Waite KA, Pilarski R, Hampel H, Fernandez MJ, et al. (2003) Germline PTEN promoter mutations and deletions in Cowden/Bannayan-Riley-Ruvalcaba syndrome result in aberrant PTEN protein and dysregulation of the phosphoinositol-3-kinase/Akt pathway. Am J Hum Genet 73: 404-411.
15. Blanchard C, Wang N, Stringer KF, Mishra A, Fulkerson PC, et al. (2006) Eotaxin-3 and a uniquely conserved gene-expression profile in eosinophilic esophagitis. J Clin Invest 116: 536-547.
16. Maxwell MA, Leane PB, Paton BC, Crane DI (2005) Novel PEX1 coding mutations and 5' UTR regulatory polymorphisms. Hum Mutat 26: 279.

17. Huang HY, Chien CH, Jen KH, Huang HD (2006) RegRNA: an integrated web server for identifying regulatory RNA motifs and elements. Nucleic Acids Res 34: W429-434.
18. Baroni TE, Chittur SV, George AD, Tenenbaum SA (2008) Advances in RIP-chip analysis : RNA-binding protein immunoprecipitation-microarray profiling. Methods Mol Biol 419: 93-108.
