# Supplementary material for: Disease-Associated Mutations That Alter the RNA Structural Ensemble
Source: PLoS Genet. 2010 Aug 19;6(8):e1001074. doi: 10.1371/journal.pgen.1001074 (PMC2924325; doi:10.1371/journal.pgen.1001074)
Supplement: Table S2 — (A) Lengths of pre- and mature mRNAs where we have identified a RiboSNitch. (B) SNPFold analysis of RiboSNitch in pre and mature mRNA UTRs revealing that a majority of the RiboSNitches identified affect the mature mRNA only. The * indicates an approximate p-value computed from a distribution of random sequences, due to the computational limitations of calculating the p-value for the longer (>2000) pre-mRNAs. (0.40 MB PDF) [file pgen.1001074.s007.pdf]

Table S2

A)

Gene	Refseq ID	Mature 5'UTR length	pre-mRNA 5'UTR length
AGRP	NM_001138	300	415
CYP1B1	NM_000104	402	792
CCR5	NM_000579	357	2761
AGT	NM_000029	508	3740
XRCC3	NM_005432	380	4399
SERPINA1	NM_001127704<br>NM_001002236<br>NM_001127705	533<br>554<br>551	7455
ABCA1	NM_005502	313	24476
BDNF	NM_001709	287	41865
PPARD	NM_006238	309	68530

B)

Gene	Refseq ID	HGMD Acc. #	SNP (loc. in spliced 5'UTR)	Mature mRNA Corr. Coeff.	pre-mRNA Corr. Coeff.	Mature mRNA p-value	pre-mRNA p-value
AGRP	NM_001138	CR073538	G79A	0.585	0.920	0.052	0.368
CYP1B1	NM_000104	CR032431	C118T	0.675	0.983	0.037	0.480
CCR5	NM_000579	CR084787	G310A	0.665	0.984	0.005	0.400*
AGT	NM_000029	CR971935<br>CR973338	G465A<br>A451C	0.694<br>0.765	0.999<br>0.995	0.051<br>0.089	0.876*<br>0.638*
XRCC3	NM_005432	CR057423	A65G	0.601	0.981	0.022	0.432*
SERPINA1	NM_001127704<br>NM_001002236<br>NM_001127705	CR061339	C116T	0.664<br>0.784<br>0.777	0.978	0.013<br>0.033<br>0.040	0.337*

A star (*) indicates approximate p-value using a random sequence rather than a full correlation coefficient analysis. Accurate p-values can only be estimated for sequences under 1000 nt in length due to the computational complexity of the calculation.
