# Supplementary material for: Disease-Associated Mutations That Alter the RNA Structural Ensemble
Source: PLoS Genet. 2010 Aug 19;6(8):e1001074. doi: 10.1371/journal.pgen.1001074 (PMC2924325; doi:10.1371/journal.pgen.1001074)
Supplement: Table S3 — eQTL data for common variant SNPs identified as potential RiboSNitches. A minority of the SNPs we identified affect transcriptional levels. (0.06 MB PDF) [file pgen.1001074.s008.pdf]

Table S3

Disease	Gene	HGMD Acc. #	UTR	Length	SNP	dbSNP Ref. ID	eQTL
Alteration of plasma zymogen TAFI concentration	CPB2	CR080756	3	427<br>453	T310A<br>T336A	rs1087	-
Chronic obstructive pulmonary disease	SERPINA1	CR061339	5	533<br>554<br>551	C116T	rs8004738	-
β-Thalassemia	HBB	CR900265<br>CR880076<br>CR961734	3<br><br>5	132<br><br>50	A111G<br>A113G<br>C33G	rs63751128<br>rs33985472<br>rs34135787	-<br>-<br>-
Hypertension	AGT	CR971935<br>CR973338	5	508	G465A<br>A451C	rs5051<br>rs5050	-<br>Acts in cis w/ AGT, score=5.903, [20]
Change in Detoxification ability	GSTM4	CR040571	5	314	C30G	rs1010167	Acts in cis w/ GSTM4, score =6.0734, [21]
Epith. ovarian cancer, serous type, prot., assoc.	XRCC3	CR057423	5	380	A65G	rs1799794	-
Allergy, assoc. with	RNASE3	CR067512	3	179	G16C	rs2233860	-
Alzheimer disease, association with	BDNF	CR014434	5	346	C301T	rs56164415	-
Reduced promoter activity, association with	AGRP	CR073538	5	300	G79A	rs34018897	-
Myocardial infarction, association with	THPO	CR014438	3	528	G35A	rs6141	-
Increased LDL cholesterol, association with	PPARD	CR035869	5	309	C223T	rs2016520	-
Eosinophilic oesophagitis, association with	CCL26	CR066323	3	169	T13G	rs2302009	-
Alleviation of zellweger syndrome	PEX1	CR053503	5	96	C44G	rs12386703	-
